# Supplementation of Jasmonic acid Mitigates the Damaging Effects of Arsenic Stress on Growth, Photosynthesis and Nitrogen Metabolism in Rice

**DOI:** 10.1186/s12284-024-00709-9

**Published:** 2024-04-27

**Authors:** Houneida Attia, Khalid H. Alamer

**Affiliations:** 1 Department of Biology, College of Sciences, Taif University, P.O. Box 11099, Taif, 21944 Saudi Arabia; 2 Biological Sciences Department, Faculty of Science and Arts, King Abdulaziz University, Rabigh, 21911 Saudi Arabia

**Keywords:** Oxidative stress, Jasmonic acid, Arsenic, Photosynthesis, Nitrogen metabolism, Osmolytes

## Abstract

Experiments were conducted to evaluate the role of exogenously applied jasmonic acid (JA; 0.1 and 0.5 µM) in alleviating the toxic effects of arsenic (As; 5 and 10 µM) stress in rice. Plants treated with As showed considerable decline in growth attributes like height, fresh and dry weight of plant. Arsenic stress reduced the content of δ-amino livulenic acid (δ-ALA), glutamate 1-semialdehyde (GSA), total chlorophylls and carotenoids, with more reduction evident at higher (10 µM) As concentrations, however exogenously supplied JA alleviated the decline to considerable extent. Arsenic stress mediated decline in photosynthetic gas exchange parameters, Fv/Fm (PSII activity) and Rubisco activity was alleviated by the exogenous treatment of JA. Arsenic stress caused oxidative damage which was evident as increased lipid peroxidation, lipoxygenase activity and hydrogen peroxide concentrations however, JA treatment declined these parameters. Treatment of JA improved the activity of nitrate reductase and glutamate synthase under unstressed conditions and also alleviated the decline triggered by As stress. Activity of antioxidant enzymes assayed increased due to As stress, and the supplementation of JA caused further increase in their activities. Moreover, the content of proline, free amino acids and total phenols increased significantly due to JA application under stressed and unstressed conditions. Treatment of JA increased the content of nitrogen and potassium while as reduced As accumulation significantly.

## Introduction

In contemporary era the metal pollution is increasing at an alarming rate. Arsenic (As) is one of the toxic metalloids, the concentrations of which is considerably increasing day by day due to industrialization thereby posing great threat to sustainable agriculture (Abedi and Mojiri 2020). Both natural as well as anthropogenic means contribute to As pollution to agricultural soils thereby causing great threat to human health after entering the food chain (Zhao et al. 2010). Predominantly existing forms of As are arsenite [As(III)] and arsenate [As(V)]. Arsenate is considered as analog of phosphate and therefore is easily transported through phosphate transporters making easy for it to impart adverse effects on normal growth and metabolism of plants (Abedi and Mojiri 2020). However, As(III) is considered as more mobile ad toxic due to its increased solubility (Ji et al. 2017) and predominant in rice field due to presence of anaerobic conditions (Mousavi et al. 2020). Transporters for As(III) in rice include Lsi1 and Lsi2 which are localised at the distal and proximal sides of exodermis and endodermis respectively (Ma et al. 2008; Abedi and Mojiri 2020). Among the transporters Lsi1 carries influx while as Lsi2 mediates transport arsenite to aerial parts (Ma et al. 2008). Because of the highly toxic nature, Environmental Protection Agency (EPA) and International Agency for Research on Cancer (IARC) has categorised As into group I human carcinogens. Arsenic inactivates enzymes by interacting with the sulphydryl groups of proteins and also results in over-production of reactive oxygen species (ROS) in plants (Ahmed et al. 2021). Arsenic stress reduces growth, photosynthesis and enzyme functioning by inducing oxidative burst in plants thereby hampering normal metabolism (Ahmed et al. 2021; Zhang et al. 2023).

It is established now that metal stress triggers over-production of ROS which impart toxic effects on the plant growth and metabolism thereby hamper yield potential significantly (Ahmad et al. 2018; Ahmed et al. 2021; Qin et al. 2022, 2024). It has been reported that heavy metals interfere with the photosynthetic efficiency, chlorophyll metabolism, redox homeostasis and the enzyme functioning (Per et al. 2016; Ahanger et al. 2020; Qin et al. 2022; Alamer et al. 2022; Alamer 2023). To counteract the ill effects of ROS nature has equipped plants with certain defense mechanisms that work to alleviate the damage (Singh 2022). It has been reported that plant genotypes exhibiting increased functioning of tolerance mechanisms survive the adverse effects of stresses better (Ahmad et al. 2018; Qin et al. 2022). In addition, selective uptake and translocation of toxic ions by the plants contributes significantly to metal tolerance which is regulated by efficient functioning of transporters (Verma et al. 2020). Under stresses significant modulations occur in the endogenous hormone concentrations to reprogram the response mechanisms at the whole plants level (Sehar et al. 2023).

In this connection, the application of phytohormones has been shown to impart beneficial effects on the stress tolerance mechanisms (Per et al. 2016; Ahmad et al. 2018; Ahmed et al. 2021; Sehar et al. 2023). Jasmonic acid (JA) is an important phytohormone which regulates several plants processes including germination, enzyme functioning, photosynthesis, osmolyte synthesis and stress tolerance (Per et al. 2016; Ahmad et al. 2016, 2017; Mousavi et al. 2020; Yadav and Singh 2023). Alleviation of damaging effects of Cd (Per et al. 2016), Ni (Sirhindi et al. 2016), salinity (Ahmad et al. 2017; Noor et al. 2022), As (Mousavi et al. 2020) and drought (Wang et al. 2021; Meng et al. 2023) has been reported in several crop species.

Rice (*Oryza sativa*) is an important food crop grown worldwide. As a staple food for majority of world population it serves as a source of protein and dietary energy for major percentage of population. On other hand global climate leading to significant water shortage farmers are compelled to use polluted water for irrigation of their rice field. The use of polluted water can affect rice growth and productivity significantly thereby posing challenge to sustainable food production. Given the importance present study was carried to analyse the influence of applied JA on the growth, nitrogen metabolism and tolerance mechanisms in response to As stress.

Rice (*Oryza sativa*) is an important food crop plant widely consumed as staple food throughout the world.It is rich in proteins and other important beneficial ingredients. Metal pollution results in significant decline in the growth and development of rice reflecting in significant reduction in yield productivity. Rice can accumulate considerable concentrations of As due to presence of phosphate transporters and aquaglyceroporins thereby affecting its growth and productivity (Khan et al. 2022). Treatment of phytohormones has been reported to alleviate the metal induced alterations in the growth and metabolism of crop plants under metal stress. In this backdrop, influence of jasmonic acid supplementation in alleviating the damaging impacts of arsenic in rice. Influence on growth, photosynthesis, antioxidant system and nitrogen metabolism wasinvestigated.

## Materials and Methods

Seeds of rice (*Oryza sativa* L. cultivar Gizza 177) were disinfected by 0.01% HgCl_2_ for 5 min. Sterilised seeds were washed five times with distilled water. Seeds were blot dried and were incubated at 28 ^o^C under dark for four days to allow germination. Thereafter, uniformly germinated seeds were grown hydroponically by transferring them to culture boxes (1 L) filled with nutrient solution and were allowed to grow for one week. Nutrient solution used was the Yoshida medium and details are outlined in Sharma et al. (2018). Nutrient solution was replaced on every alternate day. To start As stress, sodium arsenite (AsIII; NaAsO_2_) at the concentration of 0, 5 and 10 µM was added to nutrient solution. In addition, treatment of methyl jasmonate (JA) in the concentration of 0.1 and 0.5 µM was also started alone as well as in combination with As. Treatment of As and JA continued for another three weeks and for every treatment four pots were maintained. Pots were kept in a glass house maintained at 16 h light period with a light intensity of 350 µmol m^− 2^ s^− 1^, day/night temperature of 30/20°C and relative humidity of 60%. Detailed treatments include: (1) control, (2) 5 µM As, (3) 10 µM As, (4) 0.1 µM JA, (5) 0.5 µM JA, (6) 5 µM As + 0.1 µM JA, (7) 10 µM As + 0.5 µM JA, (8) 10 µM As + 0.1 µM JA, and (9) 10 µM As + 0.5 µM JA. Four-week-old seedlings (three weeks after treatment) were analysed for different parameters as described below:

### Plant Height, Fresh and dry Weight of Plant

Plant height was measured by using a scale. Fresh weight of whole plant was taken immediately and entire water was blot dried before recording the weight. Plants were dried in oven for 72 h at 60 ^o^C to measure the dry weight.

### Estimation of Glutamate 1-Semialdehyde (GSA), δ-Amino Levulinic acid (δ-ALA), Total Chlorophylls and Carotenoids

Method described by Kannangara and Schouboe (1985) was followed for estimating the GSA content. Fresh 200 mg leaf tissue was homogenised in 0.1 N HCl however another set from each treatment was incubated in 500 µM gabaculine under light for 4 h and followed by extraction in 0.1 N HCl. Homogenate was centrifuged at 15 000*g* followed by addition of HCl and 3-methyl-2-benzothiazolinonehydrazone (MBTH) to the supernatant. After incubating the mixture for 2 min in boiling water bath, the samples were cooled and FeCl_3_ was added. Optical density was taken at 620 nm.

For determination of δ-ALA content, two separate sets from each treatment were taken and one set was incubated in levulinic acid (60 mM) under light for 4 h while as other set was extracted immediately in sodium acetate buffer (1 M, pH 4.6). After centrifuging the extract at 15000*g*, supernatant mixed with acetyl-acetone and boiled. After cooling Ehrlich’s reagent, glacial acetic acid and perchloric acid were added and samples were thoroughly mixed. After incubating the samples for 10 min, optical density was taken at 555 nm (Harel and Klein 1972). For estimation of total chlorophylls and carotenoids fresh leaf tissue was extracted in acetone and absorbance recorded at 480, 645 and 663 nm (Arnon 1949).

### Measurement of Gas Exchange Parameters, PSII Activity (Fv/Fm) and Rubisco Activity

Photosynthetic gas exchange parameters including net photosynthetic rate (Pn), intercellular CO_2_ concentration (Ci) and stomatal conductance (gs) were measured using portable photosynthetic apparatus Li-6400 (LI-COR Inc., USA). For measuring Fv/Fm, modulated chlorophyll fluorometer (PAM 2500; Walz, Germany) was used and leaves weredark adapted for 25 min. For assaying activity of Rubisco, method described by Sharwood et al. (2016) was followed. Briefly, fresh 500 mg leaf tissue was ground in chilled extraction buffers which contained 50 mM Tris-HCl (pH 8.0), 10 mM β-mercaptoethanol, glycerol (12.5%), 1 mM EDTA, 10 mM MgCl_2_ and 1% polyvinylpyrrolidone (PVP). Homogenate was centrifuged at 15,000*g* for 10 min and supernatant was collected and used for measuring the activity of Rubisco. Assay mixture contained 10 mM Tris-HCl (pH 8.0), 10 mM MgCl_2_, 1 mM EDTA, 0.2 mM NADH, 20 mM NaHCO_3_, 5 mM dithiothreitol, 5 mM ATP, 10 U ml^− 1^ of each glyceraldehyde- 3-phosphodehydrogenase and 3-phosphoglycerate kinase, and 10 mM ribulose 1, 5-bisphosphate (RUBP). Optical density was taken at 340 nm and protein content was estimated according to Lowry et al. (1951).

### Estimation of Osmolytes

Among the osmolytes, proline and free amino acids were estimated. Proline was extracted from tissues in 3% sulphosalicylic acid and absorbance was taken at 520 nm after reacting the mixture with nihydrin reagent (Bates et al. 1973). Content of free amino acids was extracted in ethanol and supernatant was reacted with nihydrin reagent. Optical density was taken at 570 nm (Moore and Stein 1984).

### Oxidative Stress Parameters

For determination of hydrogen peroxide (H_2_O_2_), fresh leaf tissue was homogenized in trichloro acetic acid (TCA) and supernatant was mixed with potassium phosphate buffer (pH 7.0) and potassium iodide. Absorbance was measured at 390 nm (Velikova et al. 2000). For measuring the lipid peroxidation content of malondialdehyde (MDA) formed after reacting the supernatant with thiobarbituric acid was measured at 532 and 600 nm (Heath and Packer 1968). Activity of lipoxygenase (LOX; EC 1.13.11.12) was assayed according to Doderer et al. (1992) using linoleic acid as substrate. Absorbance was taken at 234 nm.

### Measurement of NR and GOGAT

Activity of nitrate reductase (NR) was measured after incubating the 300 mg freshly cut leaf tissue in 100 mM potassium phosphate buffer (pH 7.5) containing KNO_3_ and n-propanol in dark at 30 °C. After mixing the aliquot with sulphanilamide and 1-nephthylethylene diamine dihydrochloride, optical density was taken at 540 nm (Jaworski 1971). Activity of NADH-glutamate synthase (NADH-GOGAT; EC 1.4.1.14) was assayed following the method described by Lea et al. (1990) and optical density was taken at 340 nm.

### Estimation of Antioxidant Functioning

Antioxidant enzymes were extracted by macerating fresh 500 mg tissue in chilled 100 mM phosphate buffer (pH 7.8) constituted with 1% polyvinyl pyrolidine, 1 mM EDTA and 0.1 mM phenyl methyl sulfonyl fluoride (PMSF). Extraction was done in pre-chilled pestle and mortar, and homogenate was centrifuged for 15 min at 12,000*g*. Supernatant was used for enzyme assay. Bayer and Fridovich’s (1987) method was employed for assaying superoxide dismutase activity (SOD) and absorbance was taken at 560 nm. For assaying catalase activity (CAT) method described by Aebi (1984) was adopted and decline in optical density was noticed at 240 nm for 2 min. Ascorbate peroxidase activity (APX) was measured by noticing the disappearance of H_2_O_2_ at 290 nm for 3 min according to method described by Nakano and Asada (1981). Glutathione reductase (GR) was assayed following Foyer and Halliwell (1976) optical density was taken at 340 nm for 2 min. For measuring glutathione S-transferase (GST) method described in Hasanuzzaman and Fujita (2013) was followed and optical density was taken at 340 nm for 2 min. Enzyme activities are expressed as EU mg^− 1^ protein and content of protein was measured according to Lowry et al. (1951). Method of Ellman (1959) was followed for determination of reduced glutathione (GSH) and standard curve GSH was used for calculation.

### Estimation of Phenols

Method of Singleton and Rossi (1965) was followed for estimation of total phenols. Briefly, extraction of dry powdered tissue was carried in methanol and after centrifugation the supernatant was mixed with Folin–Ciocalteu reagent. Optical density was taken at 765 nm and gallic acid was used for calculation.

### Measurement of as, N and K

Arsenic content in dry tissue was estimated using inductively coupled plasma mass spectrometry (ICP-MS, Agilent 7500 USA) after digesting 100 mg tissue in HNO_3_. For estimation of nitrogen modified micro-Kzeldahl method was used (Jackson 1973) and potassium was determined flame photometrically.

### Statistical Analysis

Data presented is mean (± SE) of three replicates. Significance of data was tested by performing the Duncan’s Multiple Range Test and least significant difference (LSD) was calculated at *p* < 0.05.

## Results

**Fig. 1:**
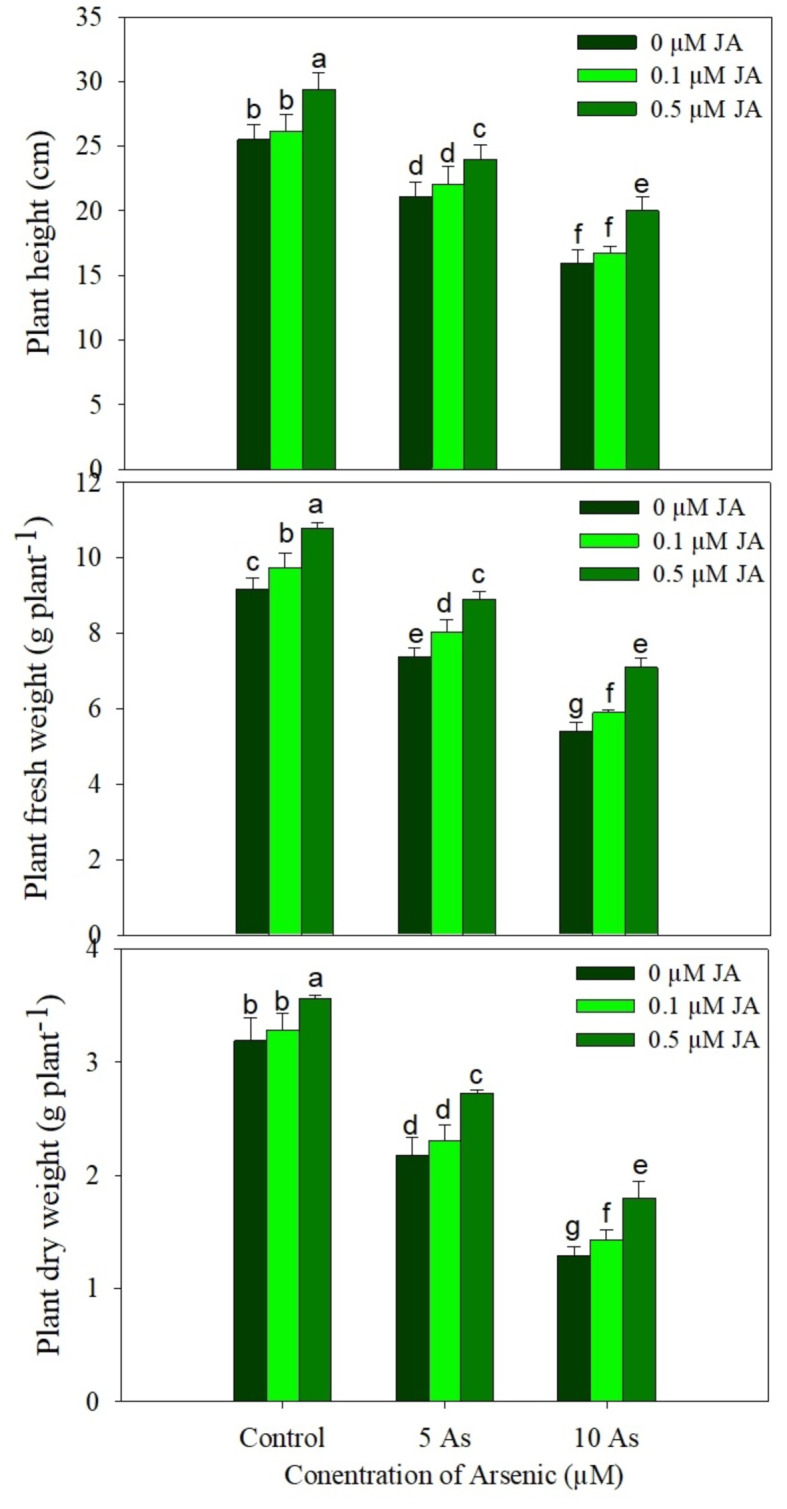
Effect of arsenic (5 and 10 µM) stress on (**A**) shoot length, (**B**) shoot fresh weight, (**C**) shoot dry weight, (**D**) root fresh weight and (**E**) root dry weight in *Oryza sativa* L. with and without exogenous supplementation of jasmonic acid (0.1 and 0.5 µM). Mean (± SE) of three replicates is presented and the different letters on bars depict the significant difference at *P* < 0.05

Growth of *Oryza sativa* was observed to increase due to supplementation of JA. However, As stress declined growth significantly. Compared to the control, parameters including plant height, fresh plant weight and dry plant weight decreased by 17.25%, 17.12% and 31.76% due to 5 µM As and by 37.49%, 41.11% and 59.57% due 10 µM As. Supplementation of JA to As stressed plants alleviated the decline considerably and the reduction of 21.52% in height, 22.68% in fresh weight and 43.86% in dry weight was observed in plants treated with 10 µM As + 0.5 µM JA over the control. In normal grown plants, treatment of 0.1 µM JA and 0.5 µM JA caused an increase of 2.35% and 15.71% in height, 6.21% and 17.55% fresh weight and 3.14% and 12.66% in dry weight respectively (Fig. 1).

**Figure legends**.

**Fig. 2:**
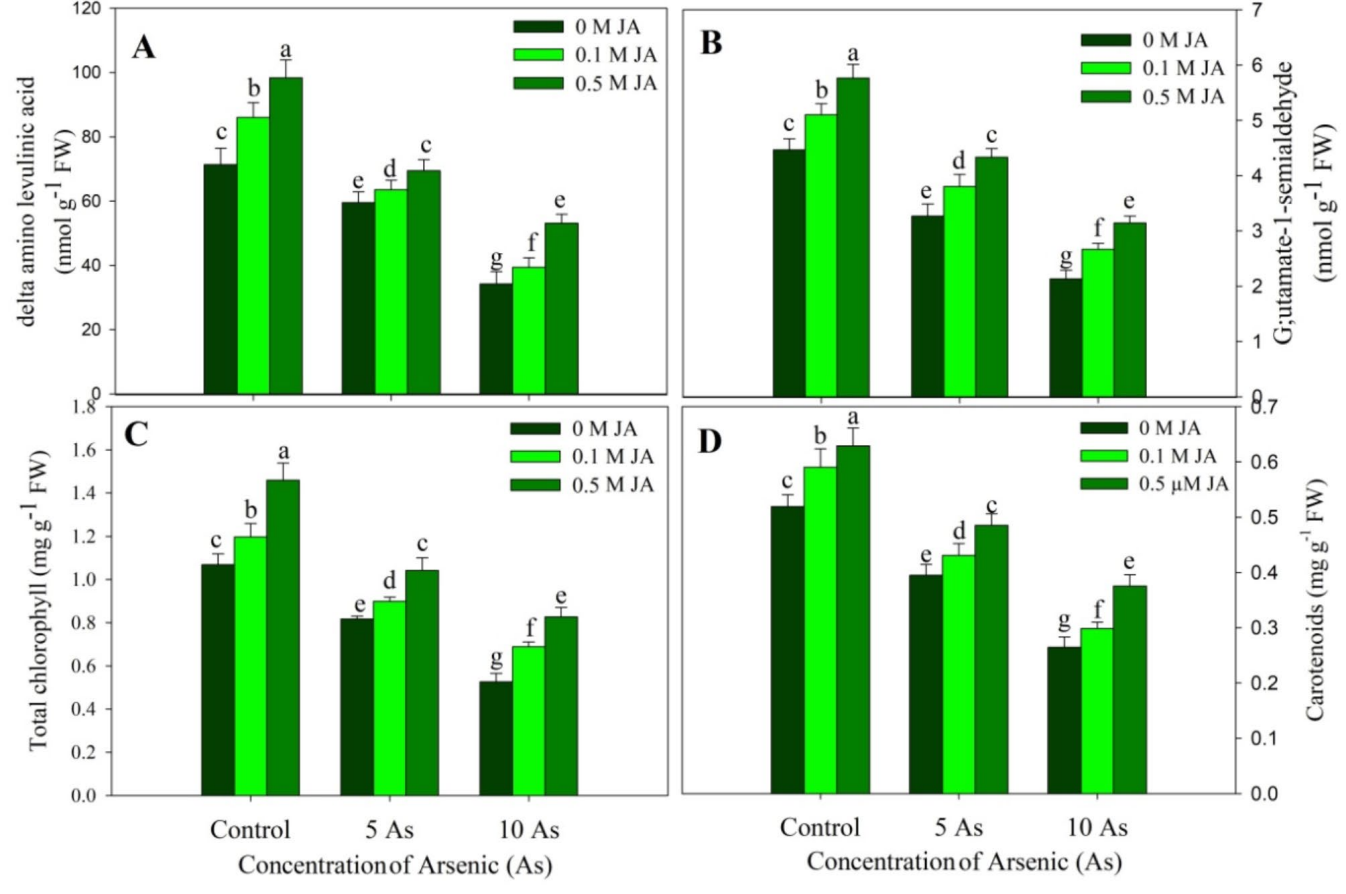
Effect of arsenic (5 and 10 µM) stress on (**A**) δ-amino levulinic acid, (**B**) gluatmate 1-semialdehyde, (**C**) total chlorophyll and (**D**) carotenoids in *Oryza sativa* L. with and without exogenous supplementation of jasmonic acid (0.1 and 0.5 µM). Mean (± SE) of three replicates is presented and the different letters on bars depict the significant difference at *P* < 0.05

Plants stressed with As showed a significant decline in the intermediate compounds of chlorophyll synthesis pathway and the photosynthetic pigments while as supplementation of JA alleviated the decline to considerable levels. The decline in content of δ-ALA (δ-aminolevulinic acid), GSA (glutamate semialdehyde), total chlorophylls and carotenoids was maximum due to 10 µM As with a percent reduction of 52.01%, 53.63%, 50.40% and 49.03% respectively over the control counterparts. Application of JA at both concentrations resulted in increased δ-ALA, GSA, total chlorophylls and carotenoids over the control. Supplementation of 0.5 µM to As stressed plants alleviated the reduction caused in δ-ALA, GSA, total chlorophylls and carotenoids significantly. As compared to control, the decline observed was only 25.59%, 28.63%, 22.64% and 27.65% in 10 µM As + 0.5 µM JA treated plants (Fig. 2).

**Fig. 3:**
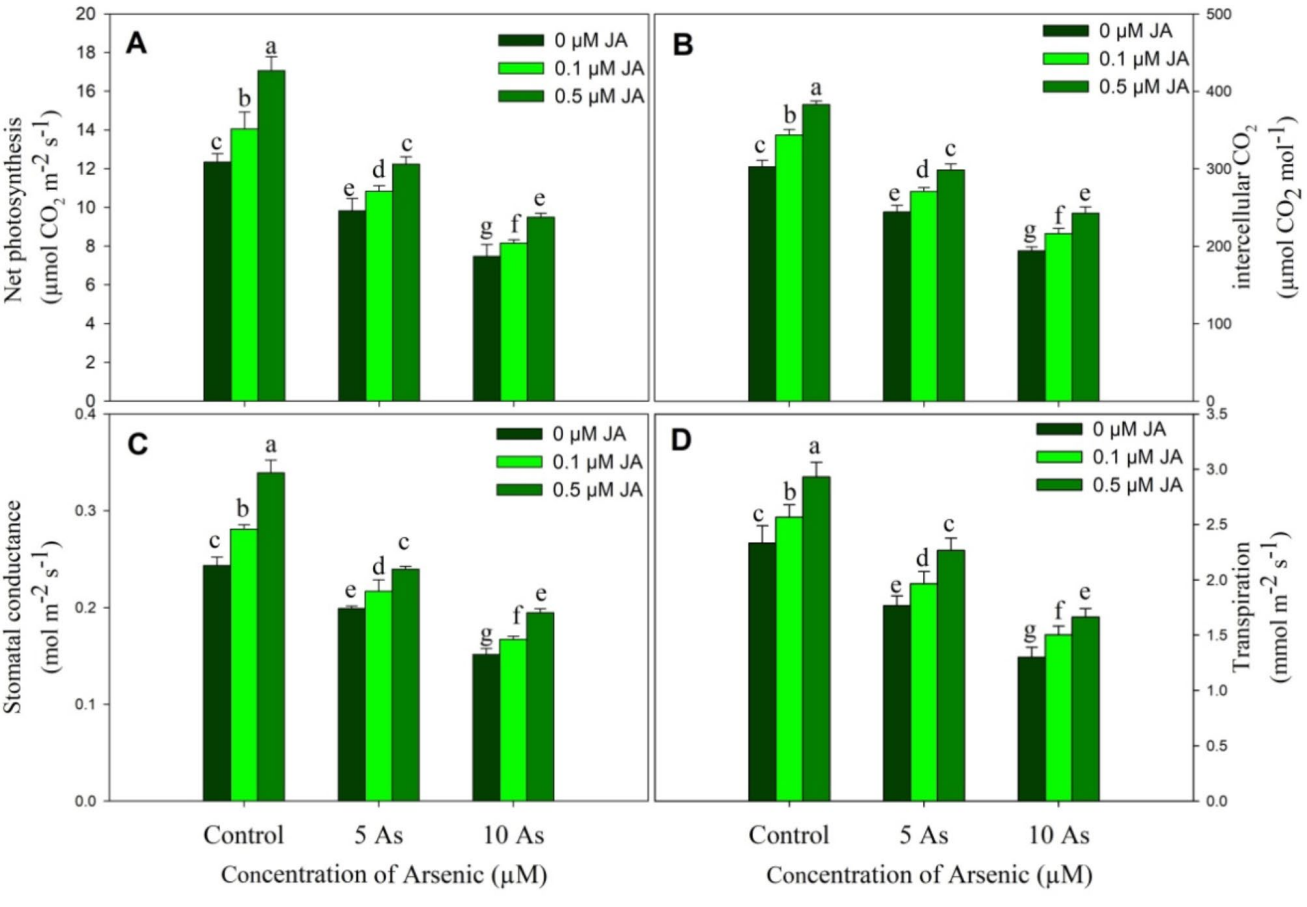
Effect of arsenic (5 and 10 µM) stress on (**A**) net photosynthesis, (**B**) interceullar CO_2_, (**C**) stomatal conductance and (**D**) transpiration in *Oryza sativa* L. with and without exogenous supplementation of jasmonic acid (0.1 and 0.5 µM). Mean (± SE) of three replicates is presented and the different letters on bars depict the significant difference at *P* < 0.05

Plants stressed with As showed considerable decline in net photosynthetic rate (Pn), intercellular CO_2_ concentration (Ci), stomatal conductance (gs) and transpiration rate (E) over the control and the decline was concentration dependent. Relative to control maximum decline of 39.49% for Pn, 35.90% for Ci, 37.77% for gs and 44.20% for E was observed in plants treated with 10 µM As. Supplementation of JA improved these parameters with 0.5 µM JA imparting highest increase and in addition alleviated the As induced decline significantly. Relative to control, decline in Pn, Ci, gs and E was only 0.81%, 1.32%, 1.52% and 3.00%, and 22.95%, 19.82%, 25.02% and 28.75% in 5 µM As + 0.5 µM JA and 10 µM As + 0.5 µM JA treated plants (Fig. 3).

**Fig. 4:**
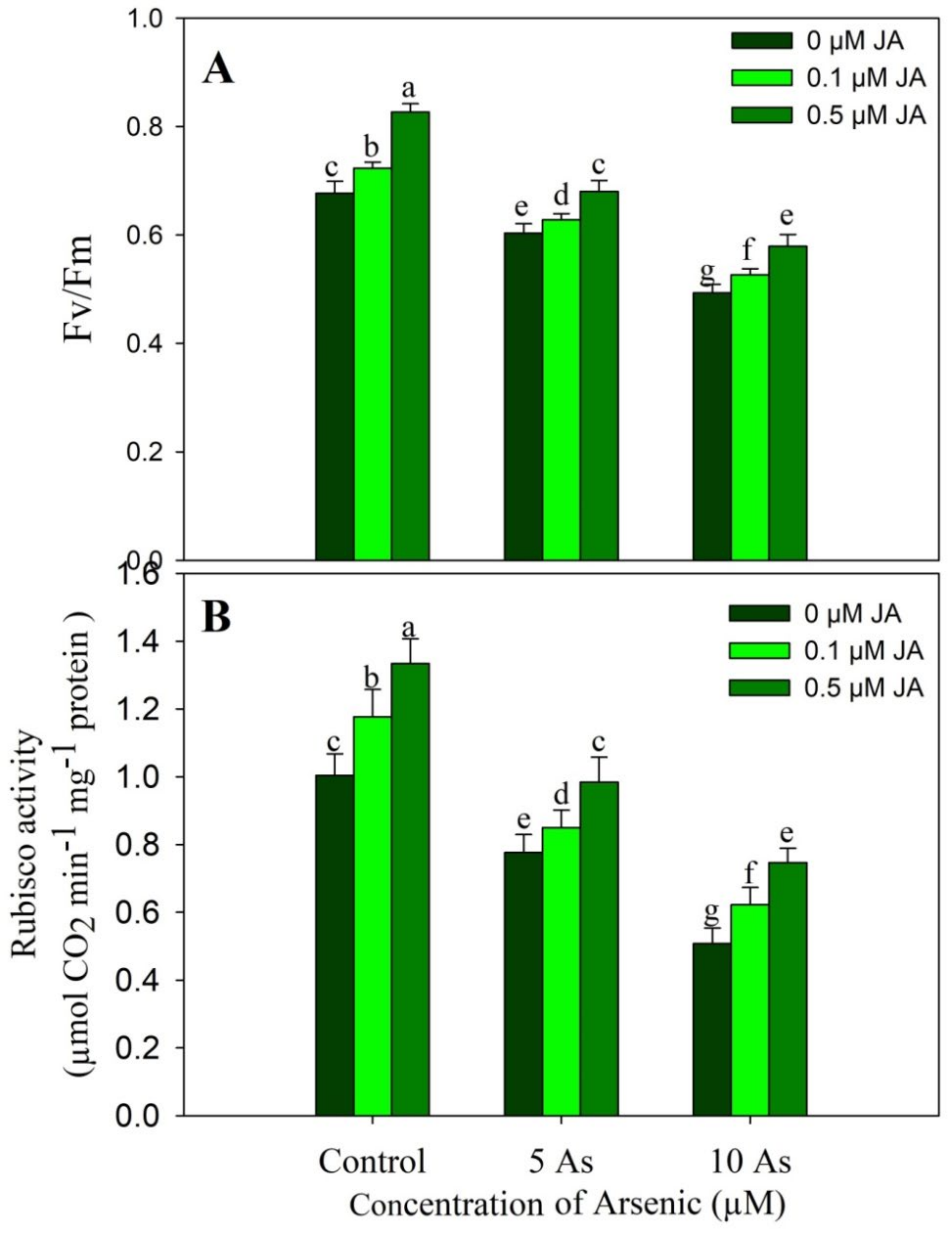
Effect of arsenic (5 and 10 µM) stress on (**A**) Fv/Fm and the activity of (**B**) Rubisco in *Oryza sativa* L. with and without exogenous supplementation of jasmonic acid (0.1 and 0.5 µM). Mean (± SE) of three replicates is presented and the different letters on bars depict the significant difference at *P* < 0.05

Arsenic stress reduced Fv/Fm and Rubisco activity over the control. Relative to control, a decline of 10.83% and 22.69% due to 5 µM As, and 27.13% and 49.40% due to 10 µM As was observed in Fv/Fm and Rubisco activity respectively. However, supplementation of JA improved the Fv/Fm and Rubisco activity significantly. Increase in Fv/Fm and Rubisco activity was 6.95% and 22.16% due to 0.1 µM JA, and 17.13% and 32.47% due to 0.5 µM JA respectively. Considerable alleviation was observed in plants treated with As + JA with maximum mitigation due to 0.5 µM JA (Fig. 4).

**Fig. 5:**
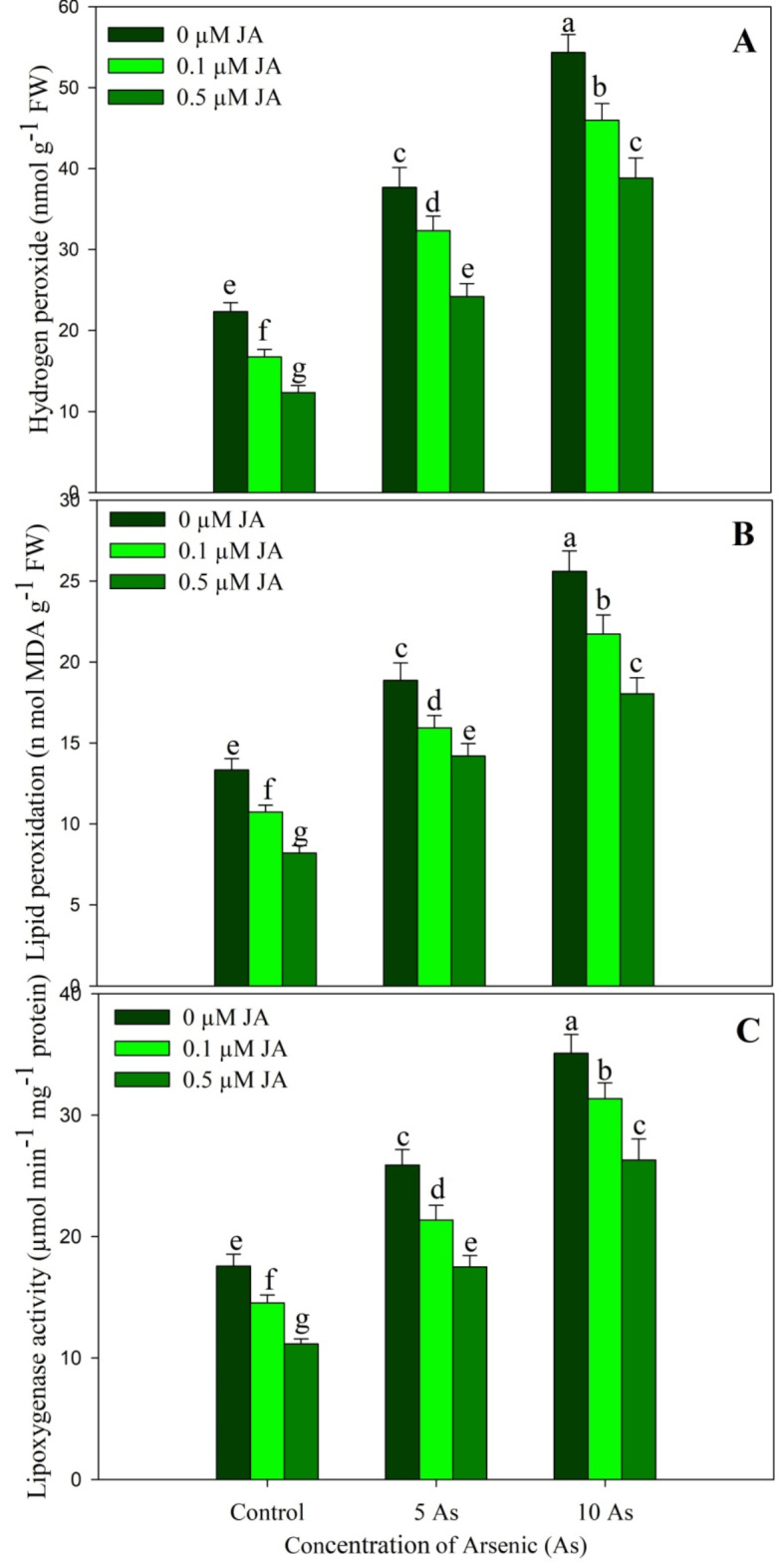
Effect of arsenic (5 and 10 µM) stress on (**A**) hydrogen peroxide, (**B**) lipid peroxidation and (**C**) lipoxygenase activity in *Oryza sativa* L. with and without exogenous supplementation of jasmonic acid (0.1 and 0.5 µM). Mean (± SE) of three replicates is presented and the different letters on bars depict the significant difference at *P* < 0.05

Plants stressed with As showed a significant increase in hydrogen peroxide (H_2_O_2_), lipid peroxidation (MDA) and lipoxygenase activity over control, however supplementation of JA decreased these attributes significantly with maximal decline of 44.78%, 38.48% and 36.44% respectively observed due to 0.5 µM JA. At the concentration of 5 µM As, increase in H_2_O_2_, MDA and LOX was 6.65%, 41.48% and 47.49% respectively while as 10 µM As resulted in increase of 143.30%, 92.04% and 99.88% respectively over control. Relative to As-stressed plants, considerable alleviation of the oxidative stress parameters was observed in As + JA-treated plants. Compared to the control, the increase in H_2_O_2_, MDA and LOX was only 73.89, 35.25% and 49.77% in 10 µM As + 0.5 µM JA treated plants (Fig. 5).

**Fig. 6:**
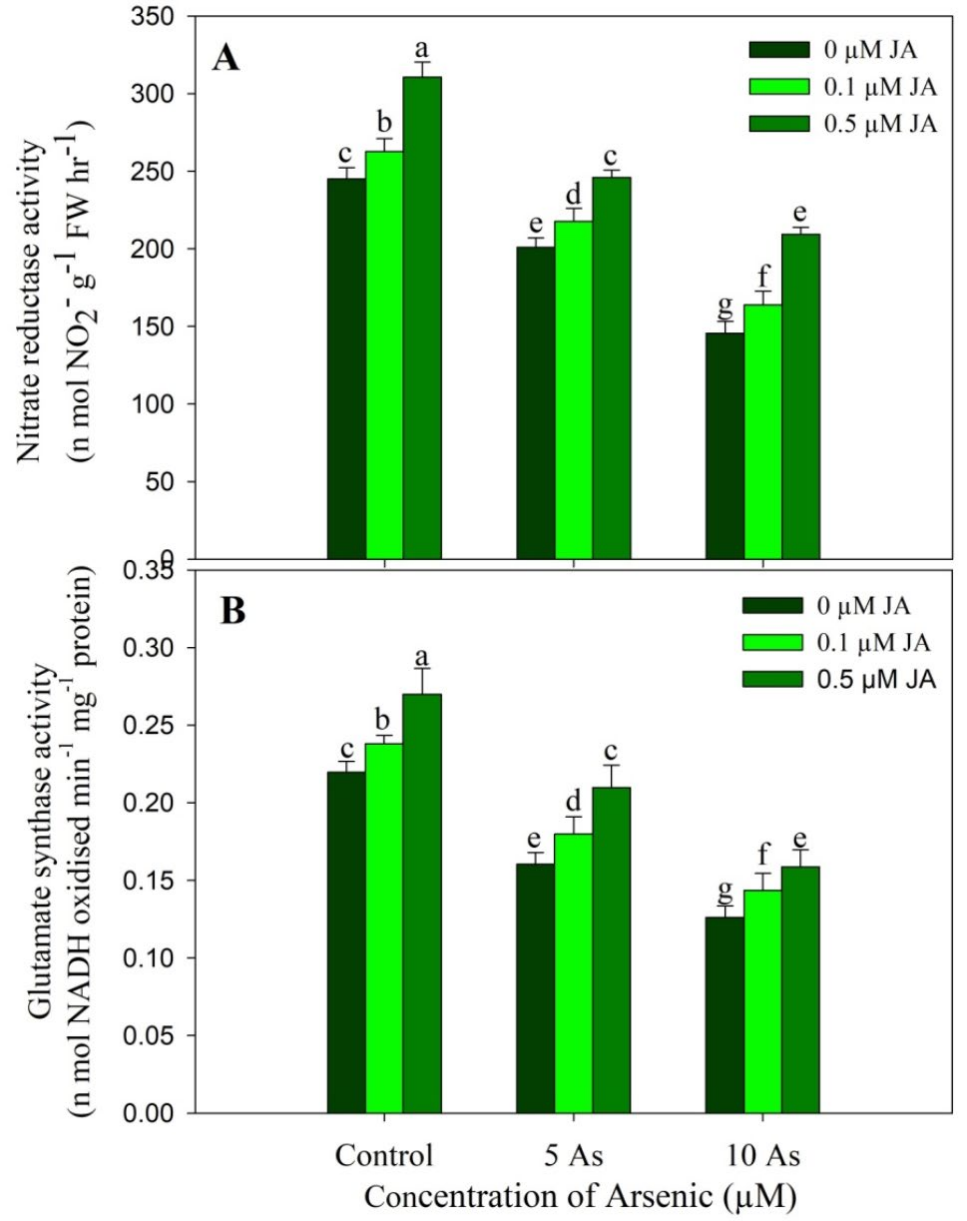
Effect of arsenic (5 and 10 µM) stress on the activity of (**A**) nitrate reductase and (**B**) glutamate synthase in *Oryza sativa* L. with and without exogenous supplementation of jasmonic acid (0.1 and 0.5 µM). Mean (± SE) of three replicates is presented and the different letters on bars depict the significant difference at *P* < 0.05

Treatment of As significantly reduced the activity of NR and GOGAT at both concentrations. Decline in NR activity was 17.95% and 41.81% while as GOGAT activity was reduced by 26.92% and 42.55% due to 5 µM and 10 µM As respectively over control. Jasmonic acid (JA) supplementation increased the activity of NR and GOGAT with highest increase observed in plants treated with 0.5 µM JA. Treatment of 0.5 µM JA caused an increase of 26.77% and 22.96% in the activity of NR and GOGAT respectively over the control. Arsenic stress induced reduction in the activities of NR and GOGAT was alleviated by the supplementation of JA. Compared to control, a decline of only 14.69% (for NR) and 27.74% (for GOGAT) was observed in plants treated with 10 µM As + 0.5 µM JA (Fig. 6).

**Fig. 7:**
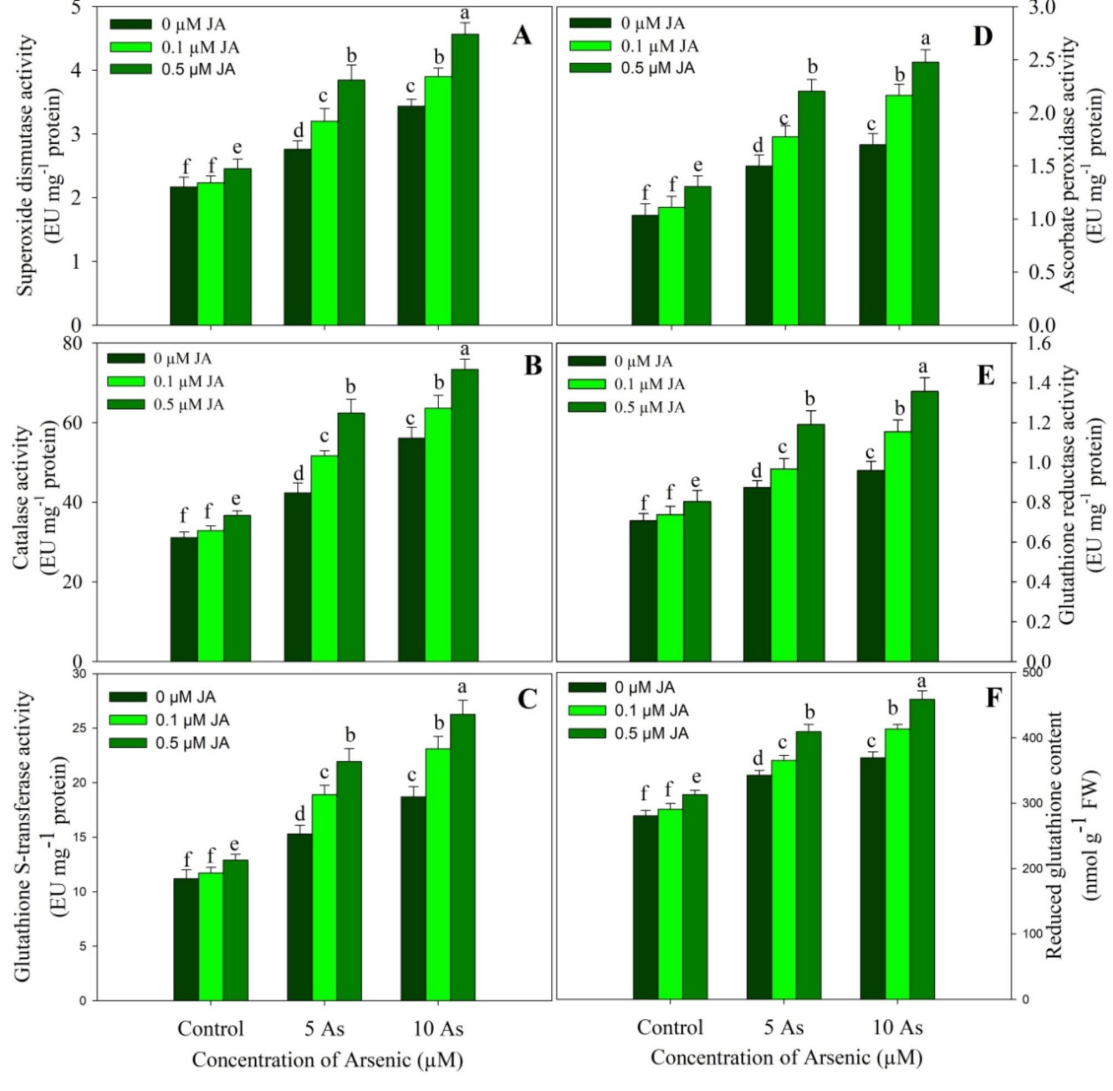
Effect of arsenic (5 and 10 µM) stress on activity of (**A**) superoxide dismutase, (**B**) catalase, (**C**) glutathione S-transferase, (**D**) ascorbate peroxidase, (**E**) glutathione reductase and the content of (**F**) reduced glutathione in *Oryza sativa* L. with and without exogenous supplementation of jasmonic acid (0.1 and 0.5 µM). Mean (± SE) of three replicates is presented and the different letters on bars depict the significant difference at *P* < 0.05

Plants grown under As stress exhibited a significant increase in the activities of antioxidant enzymes assayed and the content of reduced glutathione. Relative to control, arsenic stress resulted in a maximum increase of 58.79% (SOD), 66.97% (GST), 80.38% catalase (CAT), 64.07% (APX), 35.68% (GR) and 31.59% (GSH) at 10 µM concentration. Supplementation of JA at 0.5 µM to unstressed plants increased the activity of SOD by 13.42%, GST by 15.17%, CAT by 18.00%, APX by 26.69%, GR by 23.42% and the content of GSH by 11.52% as compared to control. Supplementation of JA to As stressed plants caused further increase in the activities of antioxidant enzymes and the GSH content achieving maximum values in 10 µM As + 0.5 µM JA treated plants. Relative to control, an increase of 111.11% for SOD, 134.46% for GST, 136.01% for CAT, 139.80% for APX, 91.71% for GR and 63.92% for GSH was observed due to 10 µM As + 0.5 µM JA (Fig. 7).

**Fig. 8:**
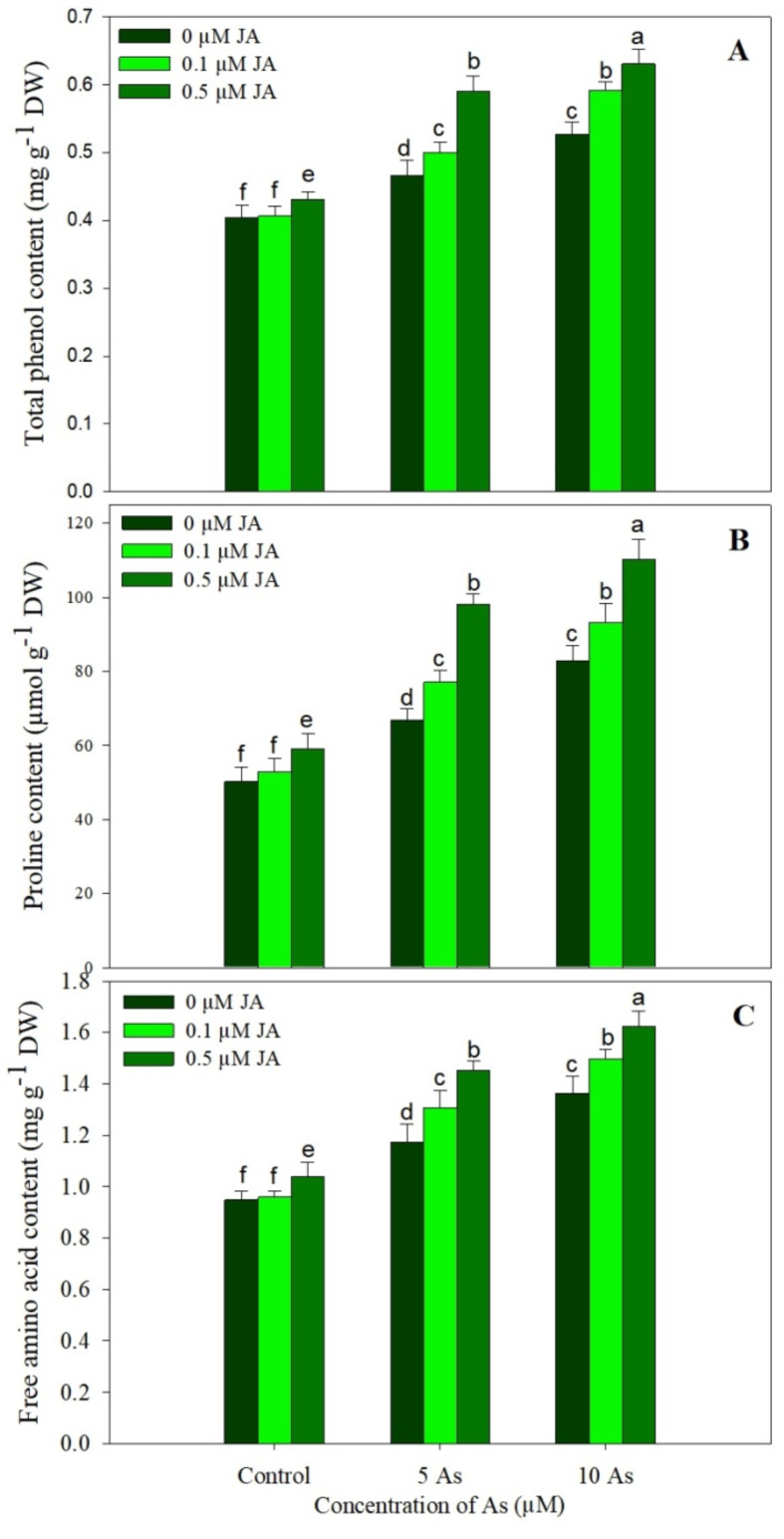
Effect of arsenic (5 and 10 µM) stress on the content of (**A**) total phenols, (**B**) proline and (**C**) free amino acids in *Oryza sativa* L. with and without exogenous supplementation of jasmonic acid (0.1 and 0.5 µM). Mean (± SE) of three replicates is presented and the different letters on bars depict the significant difference at *P* < 0.05

Phenols showed increased accumulation due to As stress with 15.18% and 30.41% increase due to 5 µM and 10 µM concentrations over control. Supplementation of JA increased phenols in unstressed plants maximally by 6.48% due to 0.5 µM JA over control. The highest increase of 56.15% was observed in plants treated with 10 µM As + 0.5 µM JA (Fig. 8A). Plants stressed with As showed an enhancement in the content of free amino acids and proline (Fig. 8B-C). Relative to control, an increase of 32.76% in proline and 23.49% free amino acids was seen in plants stressed with 5 µM As while as plants treated with 10 µM As showed an increase of 64.70% and 43.61% respectively. Supplementation of JA increased the proline and free amino acids under unstressed condition and caused further increase in their accumulation when applied to As stressed plants. Maximal increase of 118.99% and 71.48% in proline and free amino acids was observed in plants treated with 10 µM As + 0.5 µM JA over the control (Fig. 8B-C).

**Fig. 9:**
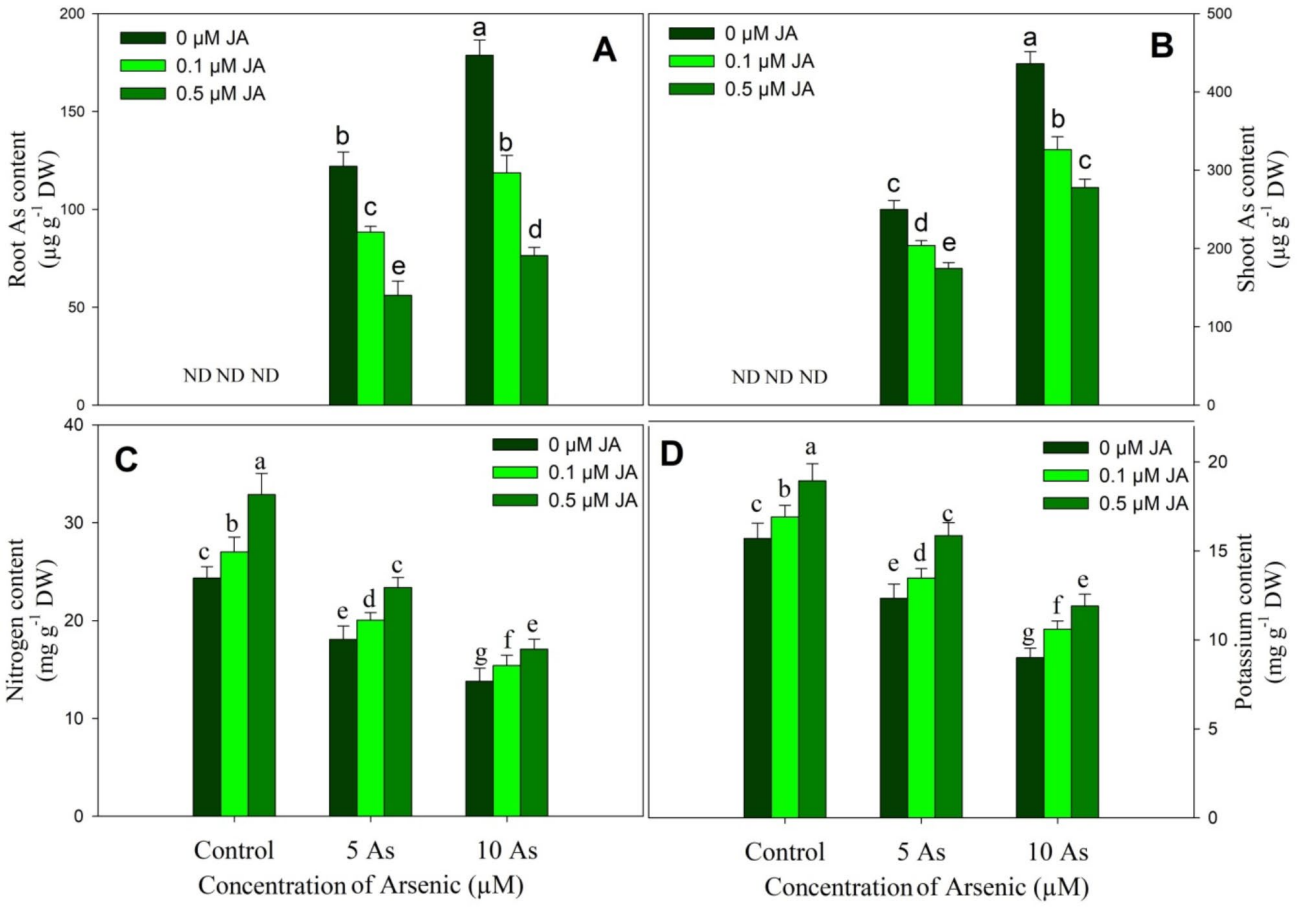
Effect of arsenic (5 and 10 µM) stress on content (**A**) root arsenic, (**B**) shoot arsenic, (**C**) nitrogen and (**D**) potassium in *Oryza sativa* L. with and without exogenous supplementation of jasmonic acid (0.1 and 0.5 µM). Mean (± SE) of three replicates is presented and the different letters on bars depict the significant difference at *P* < 0.05

Supplementation of JA to As stressed plants reduced the accumulation of As significantly in shoot and root of rice plants and reduction was much evident due to 0.5 JA supplementation. Decline in As content was 27.59% and 33.58% in root, 18.47% and 25.22% in shoot in plants treated with 5 µM As + 0.1 µM JA and 10 µM As + 0.1 µM JA respectively over the arsenic stressed plants. However, highest reduction in As was 54.09% and 57.11% in root and 30.12% and 36.31% in shoot due to 5 µM As + 0.5 µM JA and 10 µM As + 0.5 µM JA treatments (Fig. 9A-B). Nitrogen and potassium declined by 25.77% and 21.41% due to 5 µM As and by 43.27% and 42.63% due to 10 µM As respectively over the control plants. In unstressed plants, supplementation of JA increased nitrogen and potassium with greater increase due to 0.5 µM concentration. Supplementation of JA to As stressed plants alleviated the decline in nitrogen and potassium with obvious alleviation observed due to 0.5 µM JA (Fig. 9C-D).

## Discussion

Metal pollution is among the serious concern that threatens the sustainable food production. Rice is important food crop and arsenic (As) stress adversely affects its growth by reducing the key metabolic functioning. In present study it was envisaged that supplementation of JA can alleviate the ill effects of As in rice and it was observed that treatment of JA considerably alleviated the As induced decline in growth parameters including length, fresh weight and dry weight of whole plant. It was observed that treatment of 0.5 µ M JA proved much beneficial in alleviating the decline caused by As stress. Earlier decline in growth of soybean (Talano et al. 2013), rice (Mousavi et al. 2020) and *Vicia faba* (Ahmad et al. 2017) due to As stress has been reported. Arsenic drastically declines the activity of apical cell meristem, cell elongation and causes mitotic aberrations thereby resulting in considerable reduction in growth of *Pisum sativum* L. (Dho et al. 2010). Jasmonic acid increases growth and biomass production in plants and improves stress tolerance (Per et al. 2016). Pretreatment of JA has been reported to reduce the toxic effects of nickel in chickpea (Sirhindi et al. 2016). In rice Mousavi et al. (2020) have also demonstrated amelioration of As induced growth decline due to JA. Increased growth due to JA supplementation can be due to enhanced uptake of beneficial elements like N and K, reduced As uptake, decreased ROS and increased antioxidant functioning. Treatment of JA reduced the uptake and accumulation of As by reducing the expression of As transporter genes including *Lsi1, Lsi2* and *Lsi6* reflecting in greater protection to cellular structure and the metabolic functions (Verma et al. 2020; Mousavi et al. 2020). Jasmonic acid mediated As tolerance is due to significant modulation of the transport, translocation and detoxification genes in rice (Verma et al. 2020). Plants treated with As showed significant decline in N and K thereby affecting the key functioning of cells and tissues. Both N and K are macro-elements involved in regulation of major cellular functions including enzyme activity, photosynthesis, chlorophyll synthesis and stress tolerance (Ahanger et al. 2019; Ahanger and Agarwal 2017; Iqbal et al. 2015).

Plants raised with As stress exhibited a significant decline in the contents of GSA and δ-ALA reflecting in considerable decline in chlorophyll and carotenoids. However, treatment of JA increased these parameters and also resulted in alleviation of the decline considerably with evident impact observed due to 0.5 µM JA. Decline in chlorophylls, carotenoids and photosynthetic parameters due to As stress has been reported in *Phaseolus vulgaris* (Stoeva et al. 2005), *Solanum melongena* (Singh et al. 2015) and *Vicia faba* (Ahmad et al. 2017). Arsenic stress significantly reduced the growth and photosynthetic efficiency of *Solanum melongena* by inducing significant decline in chlorophyll fluorescence parameters (Singh et al. 2015). Jasmonic acid application increases chlorophyll and photosynthetic parameters under unstressed and stressed conditions (Per et al. 2016; Sirhindi et al. 2016) however the impact of As and JA on the content of GSA and δ-ALA has not been reported. Plants exposed to metal (Qin et al. 2022) and salinity (Al-Mushhin 2022; Attia 2023) stress show apparent decline in the content of chlorophyll biosynthesis intermediates including GSA and δ-ALA resulting in reduction in chlorophyll. Stresses increase chlorophyll degradation, activating chlorophyllase (Szafranska et al. 2017) with concomitant decline in activity of biosynthetic genes (Qin et al. 2020). Supplementation of JA may have increased the expression of genes coding the enzymes involved in chlorophyll synthesis. The alleviatory impact of applied JA was obvious on the stomatal as well as non-stomatal attributes of photosynthesis. Earlier, application of JA at 1 µM has been reported to alleviate the decline in chlorophyll, carotenoids and Fv/Fm in As stresses rice (Mousavi et al. 2020). Verma et al. (2020) have also demonstrated alleviation of As induced decline Pn, gs and E in rice due to JA (0.25 µM) supplementation. Heavy metal toxicity drastically affects the chloroplast stability (Per et al. 2016) and brings down the photosynthetic efficiency, however phytohormone supplementation has been reported to impart beneficial effect leading to improved photosynthesis and growth (Per et al. 2016; Ahmed et al. 2021). In addition, supplementation of JA increased the activity of Rubisco while as arsenic stress resulted in significant decline. Rubisco is main enzyme in carbon fixation and stress induced alteration in its activity can influence the entire metabolism and growth (Mo et al. 2016). Photosynthetic efficiency and the carboxylation is significantly influenced by the Rubisco activity and the functioning of stomatal parameters (Galmes et al. 2013). Phytohormones prevent the metal-induced damage to photosynthetic machinery by reducing the accumulation of toxic ROS and increasing the phytochelatin synthesis thereby reducing the chances of metal- protein interaction (Muhammad et al. 2021).

Treatment of rice seedlings with As resulted in considerable increase in ROS including H_2_O_2_ which caused significant increase in oxidative damage effects evident as increased lipid peroxidation and lipoxygenase. However, the supplementation of JA prevented the oxidative effects of As causing greater alleviation due to 0.5 µM concentrations. Excess generation of ROS due to stress factors results in peroxidation of lipids and proteins hence causing structural and functional destabilization of key macromolecules (Hasanuzzaman et al. 2020; Singh 2022). Increased accumulation of ROS including H_2_O_2_ has been reported in *Glycine max* (Chandrakar et al. 2018) and *Vicia faba* (Ahmad et al. 2017) resulting in increased lipid peroxidation. In rice genotypes, Tripathi et al. (2012) have demonstrated significant enhancement in the concentration of ROSs resulting in increased lipid peroxidation and generation of excess ROS was correlated with increased NADPH oxidase activity. Supplementation of JA have been reported to potentially reduce the oxidative effects of stresses including the metal toxicity in numerous crop plants (Per et al. 2016; Sirhindi et al. 2016; Yu et al. 2021). Supplementation of JA reduces the generation of ROSby causing significant decline in the activity of ROS generating enzymes like NADPH oxidase (Yu et al. 2021). Treatment of JA to As stressed rice reduced the ROS generation (Mousavi et al. 2020) and the lipid peroxidation (Mousavi et al. 2020; Verma et al. 2020). Alleviation of the oxidative effects of As Increased lipoxygenase activity due to metal stress and its subsequent decline due to exogenous supplementation of protectants like phytohormones reflects the prevention of the oxidative effects of stresses (Nahar et al. 2016; Ahanger et al. 2020).

Plants exhibiting decline in oxidative effects under stresses has been ascribed to up-regulation of the mechanisms aimed to neutralise the excess ROS (Soliman et al. 2020; Ahanger et al. 2019, 2020; Qin et al. 2022). Among the ROS neutralising pathways antioxidant system has the key role and in present study the functioning of antioxidant system was significantly up-regulated by the exogenous supplementation of JA to As stressed plants. Supplementation of JA improved the activity of antioxidant enzymes assayed and increased the content of reduced glutathione, a key non-enzymatic antioxidant. Plants exposed to metal stress show considerable enhancement in the activity of antioxidant system leading to mitigation of adverse effects of stresses (Per et al. 2016; Ahanger et al. 2020; Qin et al. 2022). It has been reported that plants exposed to As stress show increased functioning of antioxidant enzymes reflecting in alleviation of the adverse effects on growth and metabolism (Tripathi et al. 2012; Singh et al. 2017; Ahmed et al. 2021). Treatment of JA prevents the oxidative effects of metal stress on membrane functioning, photosynthesis and growth by up-regulating the antioxidant functioning (Per et al. 2016; Sirhindi et al. 2016). Improving the antioxidant functioning by exogenous application of JA to alleviate the damaging effects of As in rice has been reported earlier by Verma et al. (2020) and Mousavi et al. (2020). Glutathione S-transferase has key role in metal tolerance (Kumar and Trivedi 2018). Enhancing the functioning of ascorbate-glutathione cycle prevents the structural and functional alteration of the chloroplast and mitochondria (Ahanger and Agarwal 2017). Reduced glutathione is an important redox component and mediates functioning of certain essential enzymes including GR and glyoxylases to counter the damaging effects of stresses (Ahmed et al. 2021). Treatment of JA increased the activity of antioxidant enzymes and the GSH more evidently at higher concentrations thereby protecting the structure and function of cells. Increasing the functioning of antioxidant system contributes to oxidative stress alleviation, improvement of photosynthesis and maintenance of chloroplast integrity (Per et al. 2016; Ahmed et al. 2021).

Plants treated with As were observed to show significant decline in the activity of enzymes involved in nitrogen metabolism. Reduced uptake of N was associated with significant decline in the assimilation as well, however supplementation of JA alleviated the decline to considerable levels. Activities of nitrogen metabolising enzymes directly influence the synthesis of amino acids and the stress tolerance in plants (Liu et al. 2022). Nitrogen containing compounds serve key functions in stress sensing, signalling and the tolerance in plants (Trovato et al. 2021; Ye et al. 2022). Maintaining increased uptake and assimilation of N due to JA supplementation may have alleviated the deleterious effects of As by mediating greater synthesis of amino acids and the proteins. Both nitrate reductase and glutamate synthase are important in N assimilation and stress mediated any change in their activity directly influences the metabolism and growth (Ahanger and Agarwal 2017). Nitrate reductase catalyses the rate limiting step in nitrogen metabolism while as glutamate synthase mediates the formation of glutamate. Increased activity of nitrogen metabolising enzymes due to JA treatments suggests its beneficial role in enhancing the growth and also increasing the nitrogen remobilisation during different growth stages. Arsenic adversely effects the activity of nitrogen assimilatory enzymes in rice has been reported by Mahajan et al. (2023).

Phenols showed a significant enhancement in their accumulation due to JA supplementation under As stress. Secondary metabolites including phenols have important role in plant growth and development under normal and the adverse conditions (Kumar et al. 2023). Phenols regulate growth, interact with other signalling molecules like ethylene, scavenge harmful ROS, strengthen antioxidant system and improve lignin and pigment biosynthesis (Ahanger et al. 2020; Tuladhar et al. 2021; Anjali et al. 2023). In present study JA supplementation mediated increase in accumulation of phenols could have resulted in elicitation of better responses to counter the As induced alterations in growth and metabolism. This increase in the content of secondary metabolites has been reported to be associated with significant increase in the activity of enzymes involved in their synthesis (Chen et al. 2018). Influence of As with and without JA supplementation on rice phenols have not been reported yet. In addition, increase in proline and free amino acids was obvious due to application of JA. Increased proline and free amino acids help plants to counter the adverse effects of stresses (Ahanger et al. 2019). Supplementation of JA has been reported to improve content of phenol and compatible osmolytes in *Brassica rapa* to protect damaging effects of drought on photosynthesis and growth (Lone et al. 2022). Osmolytes protect plant functioning under adverse conditions by preventing the excessive accumulation of ROS and enzyme degradation, mediating stress signalling and maintaining tissue water content. Increased accumulation of proline and other amino acids due to As stress has been reported in *Spinacia oleracea* (Pavlik et al. 2010). In present study also JA treated seedlings showed increased proline and amino acid content which may have resulted in reduced oxidative damage and improved pigment synthesis and photosynthesis, however further studies are required to know actual mechanisms.

## Conclusion

Conclusively, arsenic reduced growth by altering the chlorophyll synthesis, photosynthesis and nitrogen metabolism. Supplementation of JA affectively alleviated the As induced damage by up-regulating the tolerance mechanisms. Improved As tolerance in JA treated plants was associated with the significant decline in the accumulation of ROS and the content of As. Therefore it is obvious that supplementation of JA can be affective in mitigating the adverse effects of As in rice by improving the chlorophyll synthesis, photosynthesis and nitrogen metabolism.

## Data Availability

The datasets used and/or analyzed during the current study are available from the corresponding author on reasonable request.

## Abbreviations

APX: ascorbate peroxidase
As: arsenic
As(III): arsenite
As(V): arsenate
CAT: catalase
Ci: intercellular CO_2_ concentration
E: transpiration rate
GOGAT: glutamate synthase
GR: glutathione reductase
Gs: stomatal conductance
GSA: glutamate 1-semialdehyde
GSH: reduced glutathione
GST: glutathione S-transferase
H_2_O_2_: hydrogen peroxide
JA: jasmonic acid
LOX: lipoxygenase activity
MBTH: 3-methyl-2-benzothiazolinonehydrazone
MDA: malondialdehyde
NADH-GOGAT: NADH-glutamate synthase
NR: nitrate reductase
Pn: net photosynthetic rate
ROS: reactive oxygen species
RUBP: ribulose 1, 5-bisphosphate
SOD: superoxide dismutase
δ-ALA: δ-Amino levulinic acid
